# Altered social and cognitive control interactions during decision-making in social anxiety

**DOI:** 10.1017/S0033291726103936

**Published:** 2026-05-18

**Authors:** Yiman Li, Nicholas T. Van Dam, Zhihao Wang, Yuejia Luo, Pengfei Xu

**Affiliations:** 1 School of Social Development, University of Health and Rehabilitation Sciences, Qingdao, China; 2 Melbourne School of Psychological Sciences, The University of Melbourne, Melbourne, Australia; 3 Center for Neurocognition and Social Behavior, Institute of Artificial Intelligence, Shenzhen University of Advanced Technology, Shenzhen, China; 4 School of Psychology, South China Normal University, Guangzhou, China; 5 Institute for Neuropsychological Rehabilitation, University of Health and Rehabilitation Sciences, Qingdao, China; 6 Faculty of Health and Wellness, City University of Macau, Macau, China; 7 Beijing Key Laboratory of Applied Experimental Psychology, National Demonstration Center for Experimental Psychology Education (BNU), Faculty of Psychology, Beijing Normal University, Beijing, China

**Keywords:** dorsolateral prefrontal cortex, social anxiety, social avoidance, social influence, temporoparietal junction

## Abstract

**Background:**

Social anxiety is characterized by fear and avoidance of social situations, yet many everyday decisions are made in the presence of others and are shaped by social influence. However, the influences of social anxiety on social decision-making and the underlying neural processes are not well understood.

**Methods:**

Fifty-five adults with varying levels of social anxiety completed a social risk decision-making task during functional magnetic resonance imaging (fMRI). In each trial, participants chose between a safe option and a risky gamble against either a human or a computer opponent, with or without information about others’ choices. Social influence on choice was quantified using repeated-measures analyses and drift–diffusion modeling, while brain activity and functional connectivity were examined using whole-brain analyses.

**Results:**

Compared to individuals with lower social anxiety, those with higher social anxiety showed reduced conformity to others’ risky choices, specifically when interacting with human, but not computer, opponents, together with a stronger starting-point bias toward safe options. These behavioral differences were accompanied by lower dorsolateral prefrontal cortex (dlPFC) activation and stronger dlPFC–temporoparietal junction (TPJ) functional connectivity.

**Conclusions:**

Social anxiety is associated with decreased social approach and reduced social influence from others in social decision contexts. Decreased activation of the prefrontal control system and its increased interactions with the social brain network point toward a conflict between heightened social monitoring and inefficient executive control. By distinguishing social-context effects from general risk aversion, this study provides a refined mechanistic framework for understanding how impaired regulatory control shapes maladaptive social decision-making in social anxiety.

## Introduction

Cooperative behavior in humans exceeds that of all other species (Melis & Semmann, 2010), the desire for which can potentially lead to the adjustment of one’s behavior or opinions to align with the majority under group pressure (Toelch & Dolan, 2015). However, individuals with high social anxiety (HSA) often experience worry and fear in social situations, especially regarding social evaluation and perceptions (Miskovic & Schmidt, 2012). Previous research on social anxiety has shown that socially anxious individuals often exhibit heightened social avoidance motivations and diminished social approach motivations (Asnaani, Rinck, Becker, & Hofmann, 2014; Hengstler, Holland, van Steenbergen, & van Knippenberg, 2014), potentially resulting from fear of negative social evaluation (Watson & Friend, 1969). Socially anxious individuals have been known to engage in risky behaviors such as alcohol or marijuana misuse (Garrison, Gilligan, Ladd, & Anderson, 2021; Terlecki & Buckner, 2015) to avoid social rejection. The strong desire to avoid disapproval and to avoid social engagement altogether are potentially at odds in social anxiety. Nevertheless, experimental findings on the relationship between social anxiety and conformity remain mixed. Some studies report no significant association, for example, non-significant links between social anxiety and conformity in ambiguous decision-making (Vargas, 2018), while others find that adolescents with HSA show context-dependent conformity, being lower under social interaction conditions but higher under social judgment conditions (Vargas, 2018; Zhang, Deng, Yu, Zhao, & Liu, 2016). In contrast, additional studies conclude that socially anxious individuals are generally more susceptible to conformity (Feng, Cao, Li, Wu, & Mobbs, 2018). Collectively, the evidence suggests that the social influence faced by HSA individuals is more complex than typically presumed. The perception of social influence is interwoven with internal social approach and avoidance motivations; enhanced susceptibility to social influence may disproportionately influence individuals with HSA.

The mechanisms underlying the interaction between social motivations and social influence remain unclear, and thus, the reasons for conflicting results are unresolved. Because social influence is omnipresent, conflicts arise when the direction of social influence contradicts an individual’s own social motivations. For example, others’ choices can influence an individual’s risk preferences; people integrate information from others and individual preferences to make decisions in a social context (Chung, Christopoulos, King-Casas, Ball, & Chiu, 2015). Therefore, it is necessary to examine how perceived social influence interacts with intrinsic social approach-avoidance motivations in social anxiety, especially when the inclination to conform leads to internal–external conflict.

While internet and computer technologies offer new avenues for connections and communications, it also presents unique challenges for individuals with social anxiety, as they may facilitate avoidance behavior. One notable challenge arises from the potential conflicts between social influence and social motivations, which can influence how individuals with HSA differentiate between interacting with humans and computers. Schultz et al. (2019) developed an innovative risk decision-making paradigm that effectively quantifies individuals’ social avoidance motivations. In this paradigm, participants make choices between safe and risky options, with the risky option involving playing a dice game with either a person or a computer (Schultz et al., 2019). They found that individuals with HSA are willing to sacrifice financial rewards to avoid humans (Schultz et al., 2019). However, in real social decision-making situations, individuals with HSA often make decisions not only according to their own social motivation and risk preference, but also under the influence of external social information.

Processing social information is associated with activity in the temporoparietal junction (TPJ; Zhang & Gläscher, 2020), while the dorsolateral prefrontal cortex (dlPFC) is involved in risk decision-making (Hiser & Koenigs, 2018; Phelps, Lempert, & Sokol-Hessner, 2014). In social decision-making, the introduction of social contexts often leads individuals to experience more emotional involvement, which can influence decision-making (Van Kleef, de Dreu, & Manstead, 2010; van’t Wout, Chang, & Sanfey, 2010). Researchers have found that the dlPFC is closely associated with decision-making strategies in social decision-making, primarily involving emotion regulation or response inhibition (Lin et al., 2022; Makwana & Hare, 2012; Steinbeis, Bernhardt, & Singer, 2012). This indicates that the dlPFC plays a role in moderating the impact of emotions induced by social information on decision-making. Furthermore, a study discussing cognitive control processes in social anxiety found that individuals with low social anxiety (LSA) engage in proactive control processes driven by dlPFC activity, whereas those with HSA additionally rely on reactive control processes driven by conflict-related dorsal anterior cingulate cortex (dACC) activity (Schmid, Kleiman, & Amodio, 2015). Therefore, we proposed that when social information influenced decision-making, differences in the ability to cope with emotional interference and conflict may lead to variations in the performance of individuals with different levels of social anxiety on the dlPFC.

This study manipulated the competitor type and others’ choices in risk decision-making, objectively measuring the social motivations of individuals with different levels of social anxiety and the extent to which they were influenced by social information. By using fMRI, we examined the neural activity underlying the way that intrinsic social motivations and external social information jointly influence decision-making in social anxiety. We hypothesized that individuals with both HSA and LSA would be influenced by others’ choices in decision-making, while individuals high in social anxiety would be more affected by social avoidance, manifesting as reduced conformity in situations where internal and external motivations conflict.

## Methods

### Participants

A total of 346 individuals signed up for the experiment (18.2% scoring >60 and 34.1% scoring <30 on the Liebowitz Social Anxiety Scale, LSAS; Liebowitz, 1987), among whom 63 were recruited for the current study. Two participants were excluded for not completing the experiment, and another six for failing the manipulation check (i.e. indicating others in the experiment were fictitious). Data from the remaining 55 participants were included in the analysis (27 males, mean age 20.2, range 18–25). Twenty-eight participants scoring above 60 were categorized into the HSA group (12 males, mean age 19.86, range 18–25), while 27 participants scoring below 30 were categorized into the LSA group (15 males, mean age 20.56, range 18–24). Previous studies have demonstrated that this method of distinguishing between HSA and LSA is effective (Rytwinski et al., 2009). All participants self-reported as healthy with no history of neurological or psychiatric disorders. The study protocol was approved by the local Institutional Review Board (Shenzhen University), and informed consent was obtained from each participant.

### Experimental procedures

The paradigm employed in this study was modified based on the research conducted by Schultz et al. (2019), requiring participants to make choices between safe and risky options. The study was a 2-group (HSA/LSA) × 2 competitor types (computer/human) × 3 others’ choices (safe influence/risky influence/mixed influence) design. Group was the between-subject factor, and the competitor type and others’ choices were within-subject factors. The experiment was a completely randomized design with a total of 216 trials and lasted for 37 min.

**Figure 1.**
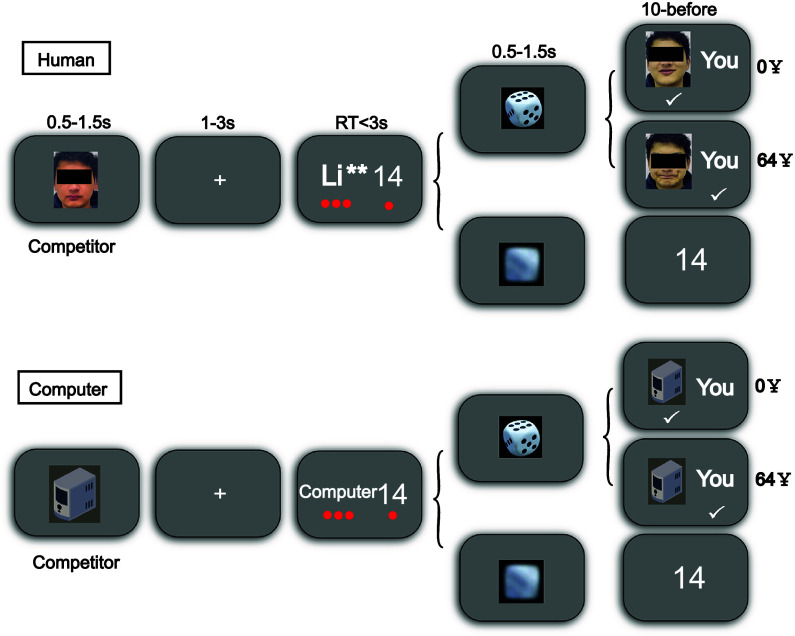
Illustration of experimental procedures. Participants were first shown the competitor type of the current round. Fixation was then presented. Subsequently, the safety option amount and the competitor’s name or ‘computer’ for the risk option were displayed. Meanwhile, the choices of four other players were indicated by small red dots (the number of dots below an option indicates how many players chose that option). After the selection was made, a video of rolling dice (risky option) or a blurred video of rolling dice (safe option) was presented. Finally, participants were presented with the outcome of the current round: If they chose the safe option, a number appeared representing the monetary reward for selecting the safe option. If they chose the risky option, a picture (dynamic expression of the competitor or a picture of the computer), along with the word ‘You’, appeared. A checkmark below the picture indicated that the opponent was the winner of the current round, while a checkmark below ‘You’ indicated that the participant was the winner of the current round.

Participants were initially presented with videos of four other ‘players’ and were informed that these four ‘players’ were randomly selected from previous game participants. The number of little red dots under the option represented how many people chose the option. The choices made by these four ‘players’ were presented to the participants as small red dots in the subsequent task. In each trial, participants were first shown the face of the current round’s competitor (Figure 1). Every participant encountered the same set of four ‘competitors’ faces, consisting of two males and two females. The facial expressions of these ‘competitors’ consistently displayed 25% disgust. Participants were then presented with a decision screen, where they were presented with two options: a risky option (a ‘name’ or ‘computer’) and a safe option (a number), as well as the choices of four other ‘players’ (represented by four small red dots). Other players’ choices consisted of (i) ‘safe influence’ trials in which at least three others selected the safe option, (ii) ‘risky influence’ trials in which at least three others chose the risky option, and (iii) ‘mixed’ trials in which the choices of four others were mixed (two safe and two risky choices). Next, the participants made their choices about whether to gamble against a competitor (risky option) or not (safe option). If the participants chose the safe option, they would receive a certain amount of money directly. If the participants choose the risky option, they would play with a competitor to receive 64 yuan if they win and 0 yuan if they lose. After choice selection, a video of rolling dice (risky option) or a blurred video of rolling dice (safe option) was presented, followed by feedback. In the end, they were presented with an angry expression on the competitor’s face while winning, and a happy expression on the competitor’s face while losing. In the condition of playing a game with a computer, all the pictures of faces were replaced with pictures of the computer host, and the names of human players were replaced with ‘computer’.

Participants engaged in approximately 5 min of practice outside the MRI. The entire experiment was conducted inside the MRI once the participants completely understood the experimental procedure. Participants were informed that the final reward was not determined by cumulating rewards from all trials but by randomly selecting the reward from one round as the ultimate payoff. Therefore, each choice made by participants could potentially determine the final reward of the experiment.

### Behavioral analysis

To quantify individuals’ social motivation in decision-making, we employed the Gaussian cumulative distribution function to estimate the Certainty Equivalent 50 (CE50). This value represents the point at which an individual has a 50% probability of choosing either option. In the present task, social approach versus avoidance motivation was indexed by the difference in CE50 between the human and computer competitor conditions. Because CE50 reflects the safe value at which participants are indifferent between the safe option and the risky option, a higher CE50 in the human condition relative to the computer condition indicates that interacting with a human opponent increases the subjective value of engaging in the gamble context. Conversely, a reduced or absent human–computer shift reflects diminished social approach tendency or relatively stronger avoidance-related decision bias in the social context. To examine how social avoidance motivation influences decision-making in HSA and LSA groups, we conducted a repeated measures analysis of variance (ANOVA) on CE50, with competitor type and others’ choices as the within-subject variables and social anxiety as the between-subject variable.

To examine the impact of others’ choices on individual selection, we conducted a repeated measures ANOVA on the proportion of safe options, with the social anxiety group and others’ choices as independent variables. To test how the competitor type and others’ choices jointly affected individuals with high and low social anxiety, we subsequently conducted a repeated measures ANOVA on the conformity, with competitor type and others’ choices as within-subject variables, and the social anxiety group as the between-subject variable. Conformity was defined as the proportion of trials in which participants chose the same option as the majority of other players, calculated separately across influence conditions. Within this framework, social influence conflict is operationalized at the condition level as situations in which the direction of others’ choices (safe vs risky) interacts with competitor type and social anxiety group to differentially shape decision patterns. Rather than being defined at the single-trial level, conflict is inferred from interaction effects, indicating competing influences of social information and individual traits on choice behavior.

### Hierarchical drift–diffusion model

To examine the theorized decision components in HSA and LSA groups, we analyzed behavioral data using the hierarchical drift–diffusion model (HDDM) implemented in the Python toolbox HDDM 0.6.0 (Wiecki, Sofer, & Frank, 2013). The standard drift–diffusion model (Ratcliff, Smith, Brown, & McKoon, 2016) decomposes choice behavior and speed into four parameters: threshold, drift rate, starting point, and non-decision time. The model represents the choices as upper and lower response boundaries, with the distance between them reflecting the threshold. Evidence accumulation is captured by the drift rate, while the starting point reflects any initial bias toward one of the two options. Non-decision time reflects the reaction time that can be allocated to basic non-decisional processes. Previous social decision-making studies have shown decision bias in different conditions; there has been a common focus on estimating the starting point bias (Biernacki et al., 2023; Ozturk et al., 2023). Therefore, this study primarily focused on starting-point bias across conditions as the main index of decision bias. To compare the differences in decision bias among different social contexts in HSA and LSA groups, we stratified the starting point structure based on competitor type, others’ choices, and the combination of competitor type and others’ choices, separately. In all three models, social anxiety was added as the intergroup variable. In addition to the starting-point-only models, we also fitted alternative models allowing the drift rate to vary to test whether evidence accumulation differences could account for the observed effects. We used the Markov Chain Monte Carlo (MCMC) fitting procedure, conducting 10,000 sampling iterations and discarding the first 1000 iterations as burn-in. Model convergence was verified through visual inspections of trace chains and convergence chains. To determine the best model, the Deviance Information Criterion (DIC) was computed to evaluate model fitting, with lower values indicating better model fit. Differences were considered meaningful when the overlap of posterior parameter distributions between groups or conditions was less than 5% (Biernacki et al., 2023). We reported the comparison of the posterior parameter distributions as *q* values instead of *p* values, indicating the proportion of the posterior parameter greater in one condition than the other.

### Neuroimaging data acquisition and preprocessing

MRI data were acquired on a 3T Siemens Trio MR system. The fMRI data were acquired using gradient echo-planar imaging (EPI) with the following parameters: TR = 1000 ms, TE = 30 ms, slices = 78, thickness = 2 mm, voxel size = 2 mm^3^, flip angle = 35°, FOV = 192 mm × 192 mm. In each of the four runs, 522 volumes were acquired. A high-resolution 3D structural brain image was acquired for each participant using a T1-weighted MPRAGE sequence: TR/TE = 2300 ms/2.26 ms, voxel size = 1 mm^3^, flip angle = 8°, FOV = 192 mm × 256 mm. Image data analysis was conducted using SPM12 (https://www.fil.ion.ucl.ac.uk/spm/). Image preprocessing, including slice-timing correction, realignment, co-registration, spatial normalization to a standard MNI template, and spatial smoothing (with a Gaussian kernel of 6 mm FWHM) was performed.

### General linear model and ROI analyses

To access the neural responses to the competitor type, we conducted the general linear model (GLM) with onsets of competitor confirmation, decision-making, and feedback in human and computer conditions as regressors. One-sample *t*-tests were conducted to assess the brain activity of the competitor type at the second level.

To explore the neural responses to the interaction between others’ choices, competitor type, and social anxiety, the onset of decision-making in human and computer trials was used as the regressor in the GLM, with the direction of others’ choices as a parametric modulation regressor. Only one parametric modulator was specified per regressor. The response of the interaction effect between competitor type and others’ choices was quantified by subtracting the brain responses to others’ choices between human and computer conditions. At the second level, a two-sample t-test was conducted to examine the differences in brain responses to the interaction effects of within-subject variables between the HSA and LSA groups.

To examine how brain activation differs between HSA and LSA groups under the influence of competitor type and others’ choices, we built another GLM, in which the onsets of decision-making in human and computer conditions were divided into safe, mixed, and risky conditions separately. Beta extraction was performed in these six conditions from the regions obtained from the interaction effects in the first GLM. Next, to explore how social anxiety, competitor type, and others’ choices interact to influence the activation, we implemented a repeated measures ANOVA with the beta weight as the dependent variable, others’ choices (safe, mixed, and risky influence), and competitor type (human and computer) as the within-subjects variable, and social anxiety as the between-subjects variable. All whole-brain results were corrected for multiple comparisons using AlphaSim correction (*p* < 0.001).

### Psychophysiological interaction (PPI) analyses

PPI analyses with the brain regions significantly activated in the interaction as the seed regions were conducted to examine modulations of others’ choices on functional connectivity in the human condition in different social anxiety groups. Blood-oxygen-level-dependent (BOLD) signals of the activated regions were extracted from the human-safe, human-mixed, and human-risky conditions. In the second level, we conducted a full factorial analysis with others’ choices as the within-subject variable and the social anxiety group as the between-subject variable. The beta values of the activated functional connectivity were extracted from the human-safe, human-mixed, and human-risky conditions. To examine how individuals with HSA and LSA were affected by others’ choices on functional connectivity in human conditions, a repeated measures ANOVA on functional connectivity was conducted, with the social anxiety group as the between-subject variable and others’ choices as the within-subject variable.

## Results

### Behavioral results

**Figure 2.**
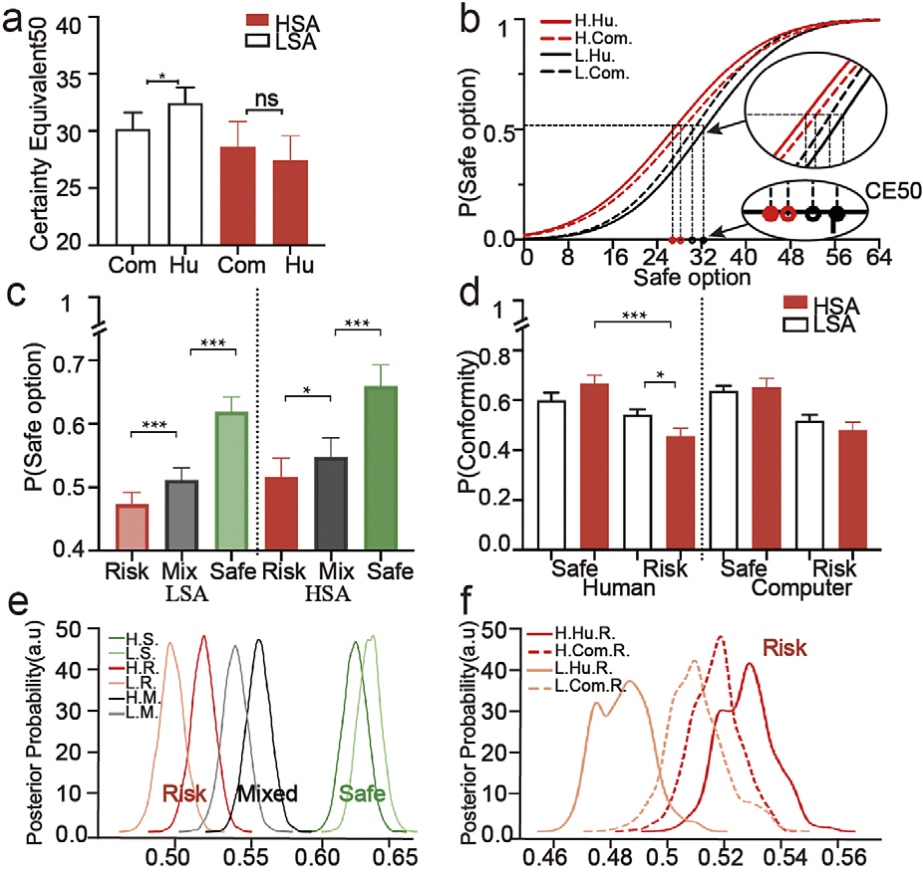
Behavior results. (a) Interaction between competitor type and social anxiety on the CE50. (b) Average cumulative distribution function curve of high and low social anxiety in different competitor type conditions. (c) Proportions of choosing the safe option in the safe influence condition, risky influence condition, and mixed condition. (d) Interaction between others’ choices, competitor type, and social anxiety on the proportion of conformity. (e) The starting point bias distribution of the HDDM model contains social anxiety and others’ choices. (f) The starting point bias distributions of HDDM models contain social anxiety, competitor type, and others’ choices: risky influence condition. *Note*: *p < 0.05; **p < 0.01; ***p < 0.001.

The repeated measures ANOVA with CE50 as the dependent variable, social anxiety group, others’ choice, and competitor type as the independent variables showed that there was a significant interaction between social anxiety and competitor type (*F* (53) = 6.527, *p* = 0.014; Figure 2a,b). Simple effects analysis revealed that the CE50 differed significantly across the competitor type in the LSA (*F* (52) = 5.597, *p* = 0.022). Specifically, individuals in LSA had significantly higher CE50 values in the human condition (32.402 ± 1.834) compared to the computer condition (30.164 ± 1.922), while there was no significant difference in competitor among the HSA (*F* (53) = 1.531, *p* = 0.221). The three-way interaction effect on CE50 was not significant (*F* (52) = 0.189, *p* = 0.829). These results indicate that competitor type modulated value evaluation in the LSA group, such that interacting with a human opponent increased the subjective value of the risky option relative to the computer condition. The absence of a difference in the HSA indicates that, compared to individuals with LSA, the HSA lack the impulse to sacrifice part of their rewards for interacting with humans.

Next, we examined the influence of others’ choices on decision-making. The results revealed a significant main effect of others’ choices (*F* (52) = 135.195, *p* < 0.001; Figure 2c). Specifically, the proportion of safe choices in the safe condition (0.639 ± 0.020) was higher than that in the mixed condition (0.530 ± 0.018), and the proportion of safe choices in the risky condition was lower than that in the mixed condition (0.494 ± 0.018). The main effect of social anxiety was not significant (*F* (53) = 1.722, *p* = 0.195). The interaction between social anxiety and others’ choices was not significant (*F* (53) = 0.569, *p* = 0.568). Regardless of the social anxiety group (HSA: (*F* (52) = 84.189, *p* < 0.001, LSA: (*F* (52) = 76.102, *p* < 0.001), others’ choices led to differences in the proportion of safe options. In both groups, the proportion of safe options chosen in the safe condition (HSA: 0.660 ± 0.029, LSA: 0.619 ± 0.029) was greater than in the mixed condition (HSA: 0.551 ± 0.025, LSA: 0.509 ± 0.025) and the risky condition (HSA: 0.523 ± 0.025, LSA: 0.465 ± 0.025). These results indicate that individuals were significantly influenced by others’ choices in decision-making regarding safe and risky influences, demonstrating a tendency toward conformity.

The repeated measures ANOVA with the proportion of conformity as the dependent variable, and social anxiety group, others’ choice, and competitor type as the independent variables revealed a main effect of others’ choices (*F* (53) = 14.902, *p* < 0.001), with greater conformity in the safe condition (0.643 ± 0.020) compared to the risky condition (0.502 ± 0.018). There was also a significant three-way interaction between others’ choice, competitor type, and social anxiety (*F* (53) = 7.752, *p* = 0.007; Figure 2d). Specifically, the interaction between social anxiety and others’ choice was significant in the human condition (*F* (53) = 4.086, *p* = 0.048), but not in the computer condition (*F* (53) = 0.524, *p* = 0.472). The results of the simple effects analysis indicated that in the human condition, individuals with HSA exhibited less conformity in the risky influence condition compared to individuals with LSA (*F* (53) = 5.212, *p* = 0.026; HSA: 0.460 ± 0.026, LSA: 0.545 ± 0.026), while there was no significant difference in the safe influence condition (*F* (53) = 2.324, *p* = 0.133). Additionally, in the human condition, individuals with HSA showed less conformity in the risky influence condition compared to the safe influence condition (*F* (53) = 15.989, *p* < 0.001; Safe: 0.670 ± 0.031, Risky: 0.460 ± 0.026), while there was no significant difference in the LSA group (*F* (53) = 1.196, *p* = 0.279). These results suggest that the joint contributions of social information and approach/avoidance motivation are associated with abnormal decision-making in social anxiety.

A sensitivity power analysis for the mixed repeated-measures design (Group × Competitor Type × Others’ Choice) indicated that with N = 55 (n = 28 and 27 per group), the study had 80% power to detect interaction effects of approximately Cohen’s f = 0.14 (partial *η^2^* ≈ 0.02), corresponding to small-to-moderate effect sizes.

To distinguish baseline risk aversion from social-context effects, we additionally compared safe-choice proportions between groups in the computer competitor condition. There was no significant group difference in safe-choice proportions (*t* = 0.59, *p* = 0.559), indicating comparable baseline risk preference across groups.

We then examined whether competitor type modulated risk preference differently across groups using a mixed repeated-measures ANOVA on safe-choice proportion, with competitor type as a within-subject factor and social anxiety group as a between-subject factor. The Group × Competitor Type interaction was statistically significant (*F* (1, 53) = 8.24, *p* = 0.006). Follow-up comparisons showed that in the HSA group, safe-choice proportion was higher in the human than in the computer condition (*t* = 3.01, *p* = 0.006), whereas the LSA group showed an opposite directional trend (*t* = −2.01, *p* = 0.055). Between-group differences were observed in the human condition (*t* = −2.02, *p* = 0.049), but not in the computer condition (*t* = 0.58, *p* = 0.562). This pattern indicates that the behavioral shift in the HSA group is specific to the human competitor context rather than reflecting generalized risk aversion.

### Hierarchical drift–diffusion model

We first fitted HDDM models in which only starting-point bias (z) was allowed to vary across group (social anxiety), competitor type, and others’ choices. Model comparison showed that Model 1 (others’ choices and social anxiety; DIC = 14,058.71; Supplementary Tables S1) exhibited a lower DIC value than Model 2 (competitor type and social anxiety; DIC = 14,396.06; Supplementary Tables S2) and Model 3 (competitor type, others’ choices, and social anxiety; DIC = 14,079.26; Supplementary Tables S3). Model 3 included all three terms, suggesting multi-way interactions. Therefore, in addition to comparing the starting point bias distributions of model 1, we also compared the starting point bias distributions in the risky influence condition of model 3 to permit understanding of possible two-way interactions.

To test whether these effects could alternatively be explained by differences in evidence accumulation efficiency, we fitted two additional models allowing the drift rate to vary. A model including both starting point and drift rate (DIC = 14,307.87) and a model including only drift rate (DIC = 30,849.68) both showed substantially worse fit than the starting point-only model (Model 3, DIC = 14,079.26). These comparisons indicate that the observed effects are primarily captured by starting-point bias rather than drift rate differences. Therefore, subsequent analyses focus on the starting point parameter.

The results of Model 1 showed a significant main effect of others’ choices on the starting bias. The starting bias was greater in the safe influence than in the mixed influence condition (*q* = 1), which, in turn, was greater than in the risky influence condition (*q* = 1). In the safe and mixed influence conditions, there was no group difference in starting bias (*q_safe_* = 0.184; *q_mixed_* = 0.918). However, in the risky influence condition, the bias distribution in the HSA group was higher (toward the safe option) than in the LSA group (*q* = 0.959; Figure 2e). In Model 3, in the risky influence condition (Figure 2f), with human competitors, the starting bias (toward the safe option) of the HSA group is higher than the LSA group (*q* = 0.999), while in the computer condition, no difference was observed between the two groups (*q* = 0.283). In the mixed and safe influence conditions, there was no such effect (Supplementary Figure S1). These findings underscore the role of others’ choices in generating differential disparities in decision bias among those with varying levels of social anxiety.

### Brain activity of competitor type

**Figure 3.**
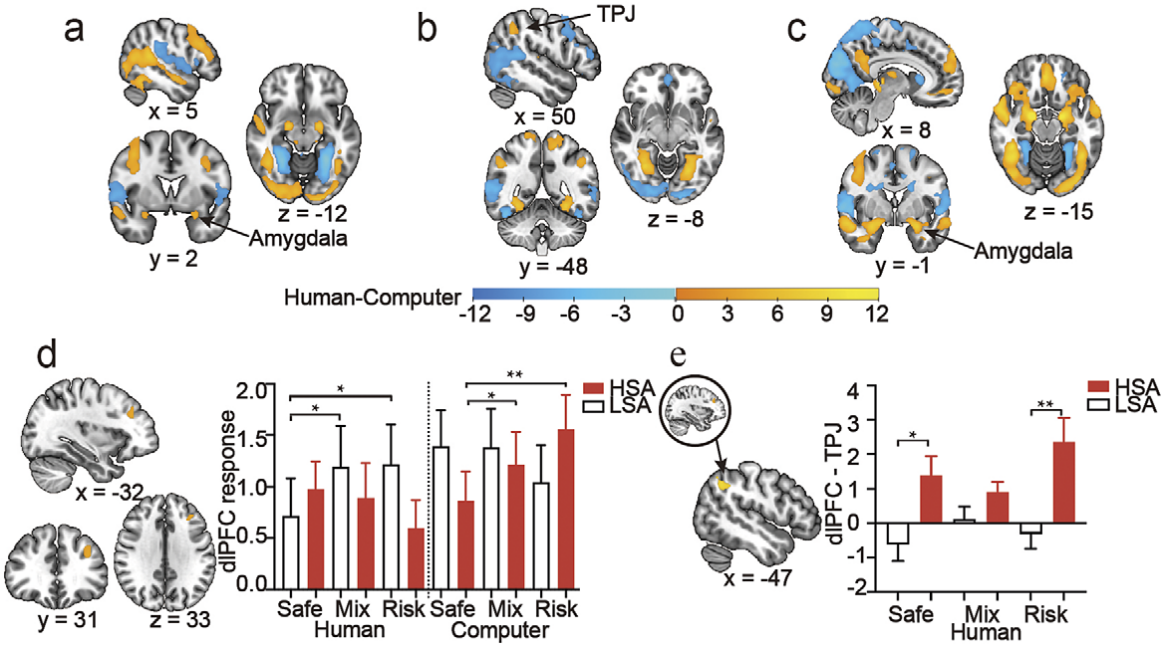
Brain activity and connectivity of competitor type and the interaction between competitor type, others’ choices, and social anxiety. Brain responses to the competitor type at the phase of (a) competitor confirmation, (b) decision-making, and (c) feedback. Warm colors indicate Human > Computer activation, and cool colors indicate Computer > Human activation. (d) Responses to the interaction between competitor type, others’ choices, and social anxiety in the decision-making phase. (e) Modulations of others’ choices and social anxiety on connectivity between left dlPFC and left TPJ in the human condition of the decision-making phase. *Note*: *p < 0.05; **p < 0.01; ***p < 0.001. AlphaSim corrected, *p* < 0.001.

Results of the activation analysis of competitor type showed that activation of the fusiform gyrus, amygdala, precuneus, and dlPFC was stronger in the human condition than in the computer condition at the competitor confirmation phase (Figure 3a; Supplementary Tables S4 and S5). In contrast, activation of brain regions such as the fusiform gyrus and inferior parietal lobules (IPL) was stronger in the computer condition. At the decision-making phase (Figure 3b; Supplementary Tables S6 and S7), the results showed stronger activation of the fusiform gyrus, superior parietal lobule (SPL), and TPJ, as well as weaker activation of the fusiform gyrus and occipital cortex in the human condition compared to the computer condition. During the feedback phase, there was stronger activation of the amygdala and orbitofrontal cortex, and weaker responses in the caudate and superior frontal gyrus in the human condition than in the computer condition (Figure 3c; Supplementary Tables S8 and S9).

### Brain activity of the interaction between competitor type, others’ choices, and social anxiety

In the analysis of the interaction, we identified a significant response of the left dlPFC to the interaction between competitor type, others’ choices, and social anxiety (Figure 3d; Supplementary Table S10). To further evaluate the robustness of this dlPFC interaction effect, given its modest cluster size, we conducted an additional small-volume correction (SVC) analysis within a dlPFC region of interest defined as an 8-mm radius sphere centered on the dlPFC peak coordinate. The effect remained significant after SVC (peak-level FWE-corrected *p* = 0.006; cluster-level FWE-corrected *p* = 0.001). The ANOVA of beta values showed a main effect of competitor type (*F* (53) = 11.365, *p* = 0.001): activation of the dlPFC was lower in the human condition (0.932 ± 0.226) than in the computer condition (1.242 ± 0.226). There was a significant three-way interaction between competitor type, social anxiety, and others’ choices (*F* (52) = 11.551, *p* < 0.001). While there were significant differences among the LSA group for the human condition (weaker activity of the dlPFC in safe vs others: *F* (52) = 4.569, *p* = 0.010; Safe: 0.715 ± 0.323, Mixed: 1.193 ± 0.369, Risky: 1.216 ± 0.334), there were no differences for the HSA group. While there were significant differences among the HSA group for the computer condition (weaker activity of the dlPFC in safe vs others: *F* (52) = 5.032, *p* = 0.010; Safe: 0.863 ± 0.314, Mixed: 1.213 ± 0.343, Risky: 1.556 ± 0.345), there were no differences for the LSA group.

We also calculated the association of the dlPFC beta values from the three-way interaction cluster with individual HDDM starting-point bias estimates. The correlation was *r* = 0.25 (*p* = 0.193), indicating only weak evidence for a direct relationship between dlPFC activation differences and starting-point bias at the individual level.

### Brain connectivity of the interaction between others’ choices and social anxiety in the human condition

PPI analysis with the left dlPFC as the seed region showed that modulations of others’ choices on functional connectivity between left dlPFC and the TPJ were different between HSA and LSA groups (Figure 3e; Supplementary Table S11). Specifically, HSA group exhibited higher functional connectivity compared to LSA group in both the safe (*F* (53) = 7.121, *p* = 0.010; HSA: 1.377 ± 0.524, LSA: −0.618 ± 0.533) and risky (*F* (53) = 10.617, *p* = 0.002; HSA: 2.365 ± 0.575, LSA: −0.311 ± 0.586) influence conditions, whereas no significant differences were observed in the mixed condition (*F* (53) = 2.909, *p* = 0.094).

To test whether dlPFC–TPJ functional connectivity scaled with the extent to which social influence modulated behavior in the human condition, we examined the association between connectivity strength and individual differences in conformity modulation. For each participant, we calculated the difference in conformity between safe and risky others’ choice conditions in the human competitor condition (safe−risky), indexing the degree to which social influence differentially shaped behavior across contexts. The correlation between dlPFC–TPJ connectivity beta values (safe−risky contrast in the human condition) and the conformity difference score was *r* = −0.34 (*p* = 0.084) in the LSA group and *r* = −0.31 (*p* = 0.104) in the HSA group. This exploratory association suggests a possible link between stronger dlPFC–TPJ coupling and reduced conformity under risky others’ choice; however, the effect did not reach statistical significance and should be interpreted with caution.

## Discussion

In this study, we attempted to resolve the competing contributions of social avoidance motivation and social conformity among those with heightened social anxiety during risky decision-making. Our findings indicate that both HSA and LSA individuals are susceptible to social influence. Notably, in HSA, social influence seemed to contribute less to behavior than avoidance-related choice tendencies when interacting with human partners. This reduction suggests a pattern consistent with reduced social approach motivation or elevated social avoidance motivation in individuals with HSA. Neuroimaging data revealed that the functional connectivity between the dlPFC and the TPJ is pivotal in this process. This connectivity may reflect altered coordination between social and cognitive control systems during social decision-making in individuals with social anxiety.

Individuals with LSA had higher CE50 in the human condition compared to the computer condition, suggesting that interacting with human players may carry additional social value for LSA individuals. However, this pattern was not observed in the HSA group. Motivation to build social relationships is one of the most universal driving forces in humans (Izuma, Saito, & Sadato, 2008; Kawamichi et al., 2016; O’Doherty et al., 2003). Social interaction serves as a form of social reward, capable of eliciting positive emotions, and is associated with the activation of the ventral striatum, a region linked to reward processing (Kawamichi et al., 2016). Similar neural representations for social rewards and monetary rewards (Izuma et al., 2008) demonstrate the rewarding nature of social interactions for general individuals. Using regression analysis, Schultz et al. (2019) found that social anxiety was positively associated with social avoidance motivation in social decision-making. In line with these studies, our results indicate that, for individuals with low social anxiety, social interaction may also serve as a reward, leading them to adjust their choices in the pursuit of social interaction. Crucially, our control analysis revealed no group difference in risk preference within the computer condition, arguing against generalized risk aversion as the primary driver. Therefore, the HSA group’s tendency toward safety in the human condition is consistent with a social-context–dependent shift that may relate to social avoidance tendencies, rather than a baseline intolerance of uncertainty.

Consistent with previous research (Chung et al., 2015), in both HSA and LSA groups, the proportion of safe choices was lowest in the risky condition and highest in the safe condition, indicating that individuals, regardless of their level of social anxiety, are influenced by external others’ choices to conform. The results of the HDDM also demonstrated the influence of others’ choices on decision-making from the starting point bias. This adjustment may stem from either identifying with others’ choices or adhering to social norms (Mahmoodi, Nili, Bang, Mehring, & Bahrami, 2022), but ultimately, this influence from others constitutes a form of social pressure.

While both groups showed conformity to others’ choices, there were important differences in behavior under different conditions between social anxiety groups. We found that HSA individuals are willing to conform to behavior that avoids risk and social interaction rather than behavior that engages risk and social interaction, suggesting an alignment of social and risk avoidance motivations. Consistent with previous studies (Chung et al., 2015; Heuer, Rinck, & Becker, 2007; Klucharev, Hytonen, Rijpkema, Smidts, & Fernandez, 2009; Schultz et al., 2019; Toelch & Dolan, 2015), our results highlight the impact of social pressure and the social avoidance motivation in individuals with HSA on social decision-making. In the human-risky condition, individuals with LSA exhibited increased conformity, likely a result of the alignment of social approach motivation and the option indicated by social pressure. For individuals with HSA, the conflict between social avoidance motivation and external social pressure led to reduced conformity. This suggests that their decision-making is driven by a prioritization of avoidance rather than a generalized reduced sensitivity to social information, as they remained responsive to social influence in the safe condition.

Besides differences in fusiform gyrus activity related to face processing between the human and computer conditions, which are consistent with previous work (Schultz & Pilz, 2009), we also observed stronger activation in the amygdala, prefrontal cortex, and TPJ, but weaker activation in the IPL in the human condition than in the computer condition. The amygdala has long been implicated in emotional cognition and social attention (Seymour & Dolan, 2008), while the amygdala–prefrontal neural pathway has been widely involved in the processing of social information, value assessment, and reward during social decision-making (Gangopadhyay, Chawla, Dal Monte, & Chang, 2021; Huang, Zucca, Levy, & Page, 2020; Tian et al., 2021). The TPJ has been shown to play an important role in the processing of social information (Decety & Lamm, 2007; Samson, Apperly, Chiavarino, & Humphreys, 2004; Santiesteban, Banissy, Catmur, & Bird, 2012), and the IPL is associated with reward probability and uncertainty in decision-making (Guo et al., 2013; Vickery & Jiang, 2009; Wang et al., 2023). Therefore, the current results suggest that more social and emotional processing is involved when people play with humans, but individuals are more focused on the uncertainty of decisions when playing with computers.

We also found that the left dlPFC plays a role in the interactive effects of others’ choices, social motivation, and social anxiety. Specifically, in the human condition, the LSA group exhibited lower left dlPFC activation in the safe influence condition than in the mixed and risky influence conditions, whereas this modulation across influence conditions was not observed in the HSA group. The dlPFC is widely recognized as a key brain region involved in cognitive control and top-down attention control (Brosnan & Wiegand, 2017; Kim, Chung, & Kim, 2013; MacDonald, Cohen, Stenger, & Carter, 2000), as well as cognitive control that supports social cognition (Weissman, Perkins, & Woldorff, 2008). The dlPFC also plays a role in balancing consideration of self and others, acting to inhibit impulsive self-centric responses in the decision-making process (Makwana & Hare, 2012). In our results, enhanced activation of the dlPFC in the risky condition reflects the engagement of cognitive control necessary to resolve the conflict between risk aversion, social influence, and social approach motivation. Consistently, individuals with social anxiety disorder have been shown to have weaker activation in the left dlPFC than controls, indicating deficits of cognitive control under stress in anxious individuals (Koric et al., 2012). In human conditions, individuals with HSA not only need to consider their own risk preference and the influence of others’ choices, but also face the burden of social information processing and the pressure of social avoidance motivation caused by human players. Therefore, we speculate that individuals with HSA may show reduced efficiency in recruiting cognitive control resources to deal with these conflicts in human conditions.

Stronger functional connectivity between the left dlPFC and TPJ in the HSA group relative to the LSA group reveals tighter coupling between cognitive control and social-information processing systems (Carter & Huettel, 2013; Saxe & Kanwisher, 2003; Schurz, Tholen, Perner, Mars, & Sallet, 2017). These findings may reflect increased reactive control or an over-exertion of social monitoring effort when interacting with human competitors in anxious individuals. This interpretation aligns with dual-mechanisms frameworks that distinguish between proactive and reactive control modes (Braver, 2012), in which reduced proactive control is accompanied by greater context-driven regulatory recruitment. Together with the reduced dlPFC activation observed in the HSA group – which suggests less efficient local control recruitment under socially demanding conditions (Brosnan & Wiegand, 2017; MacDonald et al., 2000; Weissman et al., 2008) – this pattern is consistent with evidence linking anxiety to reduced prefrontal control efficiency (Bishop, 2009; Xu et al., 2020).

Taken together, our behavioral, computational, and neural results consistently show that individuals with different levels of social anxiety differ in how their decisions are shaped by competitor type and others’ choices, suggesting that differences in social motivation may contribute to their decision patterns in social contexts. However, previous decision-making studies in clinical populations have shown that risk avoidance and social avoidance are not equivalent constructs and can arise from different mechanisms (Knowles & Olatunji, 2023; Pushkarskaya et al., 2015; Starcevic et al., 2011). It should be noted that the present task cannot directly measure internal motivational states. Although our computer control condition helps disentangle social-context effects from baseline risk aversion, choosing the safe option may still reflect complex risk-related considerations rather than social avoidance per se. Therefore, interpretations at the level of internal motivation should be made with caution, and this represents an important limitation of the current design.

In addition, although the sample size was comparable to prior fMRI studies of social decision-making, the present design was primarily powered to detect small-to-moderate interaction effects. Smaller effects, especially brain–behavior associations, may therefore be underpowered and should be interpreted with caution. Replication in larger samples will be important to establish the stability and generalizability of these effects.

In summary, this study examined the interaction between social motivations and external social influence in social anxiety within a risk decision-making framework. The results revealed that both HSA and LSA individuals are influenced by social motivation and others’ choices in their decision-making. Notably, individuals with HSA exhibit reduced conformity to risky options when interacting with humans, consistent with a tension between avoidance-related tendencies and external social pressures. This process may involve cognitive control and social cognition processes, associated with the functional connectivity between the dlPFC and the TPJ. In society, a certain degree of social approach motivation and conformity is an expectation under cooperative behavior. These demands are difficult to avoid, persist over the long term, and become a form of pressure. Therefore, when discussing social anxiety, it is crucial to consider not only social motivation but also individual attitudes toward social pressure and how to manage this pressure. Addressing these factors can provide a deeper understanding of social anxiety and inform more effective interventions.

## Supporting information

10.1017/S0033291726103936.sm001 Li et al. supplementary material Li et al. supplementary material

## Data Availability

The data and code that support the findings of this study are available from the corresponding author upon reasonable request.
