# Supplementary material for: Altered social and cognitive control interactions during decision-making in social anxiety
Source: Psychol Med. 2026 May 18;56:e156. doi: 10.1017/S0033291726103936 (PMC13200152; doi:10.1017/S0033291726103936)
Supplement: Li et al. supplementary material [file S0033291726103936sup001.docx]

**Li et al.**

**Supplementary Materials**

**
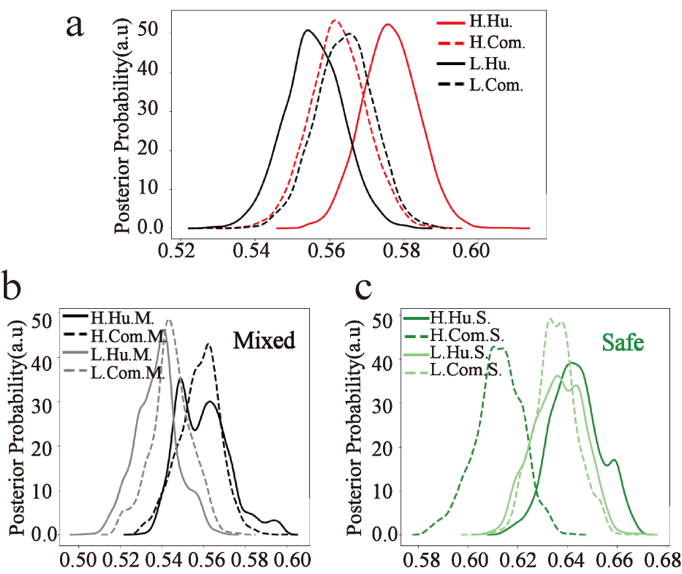
**

**Figure S1.** (a) The starting point bias distribution of the HDDM model contains social anxiety and competitor type. The starting point bias distributions of HDDM models contain social anxiety, competitor type, and others’ choices: (b) mixed influence condition and (c) safe influence condition.

**Table S1.** The starting point bias distribution in the model considers social anxiety and others’ choices.

|  | mean | std | 2.5q | 25q | 50q | 75q | 97.5q |
| --- | --- | --- | --- | --- | --- | --- | --- |
| z (H.M.) | 0.561 | 0.009 | 0.544 | 0.555 | 0.561 | 0.567 | 0.578 |
| z (L.M.) | 0.544 | 0.009 | 0.526 | 0.538 | 0.543 | 0.549 | 0.561 |
| z (H.R.) | 0.521 | 0.009 | 0.504 | 0.515 | 0.521 | 0.527 | 0.539 |
| z (L.R.) | 0.500 | 0.009 | 0.483 | 0.495 | 0.500 | 0.506 | 0.518 |
| z (H.S.) | 0.626 | 0.008 | 0.610 | 0.621 | 0.626 | 0.632 | 0.643 |
| z (L.S.) | 0.637 | 0.008 | 0.621 | 0.631 | 0.637 | 0.642 | 0.653 |

**Table S2.** The starting point bias distribution in the model considers social anxiety and competitor type.

|  | mean | std | 2.5q | 25q | 50q | 75q | 97.5q |
| --- | --- | --- | --- | --- | --- | --- | --- |
| z (H.Com.) | 0.562 | 0.008 | 0.547 | 0.557 | 0.562 | 0.568 | 0.578 |
| z (H.Hu.) | 0.576 | 0.008 | 0.562 | 0.571 | 0.576 | 0.581 | 0.591 |
| z (L.Com.) | 0.564 | 0.008 | 0.548 | 0.559 | 0.564 | 0.569 | 0.579 |
| z (L.Hu.) | 0.556 | 0.008 | 0.540 | 0.551 | 0.556 | 0.561 | 0.572 |

**Table S3.** The starting point bias distribution in the model considers social anxiety, others’ choices, and competitor type.

|  | mean | std | 2.5q | 25q | 50q | 75q | 97.5q |
| --- | --- | --- | --- | --- | --- | --- | --- |
| z (H.Com.M.) | 0.558 | 0.010 | 0.537 | 0.552 | 0.559 | 0.565 | 0.576 |
| z (H.Hu.M.) | 0.560 | 0.012 | 0.540 | 0.550 | 0.559 | 0.568 | 0.591 |
| z (L.Com.M) | 0.545 | 0.010 | 0.523 | 0.539 | 0.544 | 0.551 | 0.563 |
| z (L.Hu.M.) | 0.538 | 0.010 | 0.517 | 0.531 | 0.538 | 0.544 | 0.558 |
| z (H.Com.R.) | 0.517 | 0.009 | 0.500 | 0.511 | 0.517 | 0.522 | 0.534 |
| z (H.Hu.R.) | 0.528 | 0.010 | 0.510 | 0.521 | 0.528 | 0.534 | 0.547 |
| z (L.Com.R.) | 0.510 | 0.010 | 0.492 | 0.503 | 0.510 | 0.516 | 0.533 |
| z (L.Hu.R.) | 0.484 | 0.009 | 0.469 | 0.477 | 0.485 | 0.491 | 0.504 |
| z (H.Com.S.) | 0.612 | 0.010 | 0.592 | 0.607 | 0.613 | 0.619 | 0.631 |
| z (H.Hu.S.) | 0.643 | 0.010 | 0.624 | 0.636 | 0.643 | 0.650 | 0.663 |
| z (L.Com.S) | 0.635 | 0.008 | 0.620 | 0.630 | 0.635 | 0.641 | 0.653 |
| z (L.Hu.S) | 0.637 | 0.010 | 0.617 | 0.629 | 0.637 | 0.644 | 0.654 |

**Table S4.** Competitor confirmation: Human-Computer.

|  |  |  |  | MNI coordinates | | |
| --- | --- | --- | --- | --- | --- | --- |
| Region | Laterality | Cluster size K_E_ | Z | x | y | z |
| Fusiform Gyrus | R | 4983 |  | 42 | -46 | -20 |
| Occipital Gyrus | R |  |  | 22 | -94 | -6 |
| Occipital Gyrus | R |  | 7.50 | 20 | -86 | -4 |
| Fusiform Gyrus | L | 1389 |  | -42 | -50 | -18 |
| Occipital Gyrus | L |  | 6.84 | -14 | -96 | -10 |
| Occipital Gyrus | L |  | 6.83 | -28 | -92 | -4 |
| Middle Frontal Gyrus | R | 1882 | 6.88 | 38 | 16 | 30 |
| Inferior Frontal Gyrus | R |  | 5.68 | 44 | 30 | 18 |
| Middle Frontal Gyrus | R |  | 5.46 | 40 | 4 | 50 |
| Amygdala | R | 165 | 6.72 | 18 | -8 | -12 |
| Amygdala | R |  | 4.92 | 28 | 0 | -18 |
| Amygdala | L | 209 | 6.45 | -18 | -8 | -12 |
| Amygdala | L |  | 5.22 | -24 | 0 | -18 |
| Precuneus | R | 1601 | 5.91 | 6 | -62 | 42 |
| Precuneus | L |  | 5.82 | -10 | -56 | 46 |
| Precuneus | R |  | 5.51 | 4 | -60 | 28 |
| Thalamus | R | 342 | 5.74 | 10 | -28 | -2 |
| Thalamus | L |  | 4.72 | -16 | -30 | -2 |
| Thalamus | R |  | 4.63 | 16 | -32 | 2 |
| Inferior Frontal Gyrus | L | 495 | 5.33 | -40 | 16 | 26 |
| Middle Frontal Gyrus | L |  | 4.64 | -34 | -2 | 44 |
| Middle Frontal Gyrus | L |  | 4.51 | -36 | 4 | 36 |
| Posterior Superior Temporal Sulcus | L | 337 | 4.72 | -54 | -58 | 10 |
| Posterior Superior Temporal Sulcus | L |  | 4.52 | -52 | -46 | 14 |
| Occipital Gyrus | L | 142 | 4.69 | -50 | -74 | 8 |
| Angular Gyrus | L |  | 3.95 | -40 | -58 | 18 |
| Angular Gyrus | L |  | 3.52 | -48 | -68 | 16 |
| Superior Parietal Lobule | R | 223 | 4.34 | 32 | -54 | 44 |
| Angular Gyrus | R |  | 3.98 | 38 | -62 | 38 |
| Angular Gyrus | L | 163 | 4.28 | -32 | -58 | 48 |
| Superior Parietal Lobule | L |  | 3.78 | -26 | -48 | 38 |
| Angular Gyrus | L |  | 3.58 | -30 | -64 | 38 |

**Table S5.** Competitor confirmation: Computer-Human.

|  |  |  |  | MNI coordinates | | |
| --- | --- | --- | --- | --- | --- | --- |
| Region | Laterality | Cluster size K_E_ | Z | x | y | z |
| Fusiform Gyrus | L | 9227 |  | -26 | -46 | -14 |
| Fusiform Gyrus | R |  |  | 26 | -48 | -14 |
| Occipital Gyrus | L |  | 7.13 | -32 | -92 | 16 |
| Inferior Parietal Lobule | R | 2627 | 6.18 | 54 | -24 | 18 |
| Precentral Gyrus | R |  | 5.43 | 58 | 6 | 4 |
| Postcentral Gyrus | R |  | 5.26 | 56 | -14 | 8 |
| Inferior Parietal Lobule | L | 1638 | 5.00 | -50 | -26 | 14 |
| Inferior Parietal Lobule | L |  | 4.95 | -58 | -28 | 14 |
| Inferior Parietal Lobule | L |  | 4.86 | -52 | -32 | 24 |
| Superior Parietal Lobule | R | 369 | 4.19 | 18 | -54 | 72 |
| Superior Parietal Lobule | R |  | 4.07 | 32 | -42 | 64 |
| Superior Parietal Lobule | R |  | 3.79 | 24 | -38 | 72 |

**Table S6.** Decision-making: Human-Computer.

|  |  |  |  | MNI coordinates | | |
| --- | --- | --- | --- | --- | --- | --- |
| Region | Laterality | Cluster size K_E_ | Z | x | y | z |
| Cuneus | L | 9249 | 6.92 | -28 | -62 | -4 |
| Fusiform Gyrus | R |  | 6.85 | 28 | -56 | -10 |
| Fusiform Gyrus | L |  | 6.45 | -28 | -52 | -12 |
| Superior Parietal Lobule | L | 481 | 5.59 | -16 | -50 | 60 |
| Superior Parietal Lobule | L |  | 5.07 | -24 | -42 | 58 |
| Superior Parietal Lobule | L |  | 4.25 | -24 | -44 | 66 |
| Superior Frontal Gyrus | L | 128 | 5.00 | -14 | 22 | 60 |
| Inferior Parietal Lobule | R | 131 | 4.89 | 48 | -46 | 38 |
| Inferior Parietal Lobule | L | 255 | 4.57 | -40 | -42 | 22 |
| Inferior Parietal Lobule | L |  | 4.45 | -44 | -46 | 40 |
| Inferior Parietal Lobule | L |  | 4.15 | -40 | -32 | 18 |
| Superior Temporal Gyrus | R | 136 | 4.57 | 62 | -12 | 2 |
| Middle Temporal Gyrus | R |  | 3.92 | 70 | -18 | -2 |
| Superior Temporal Gyrus | R |  | 3.65 | 52 | -20 | 6 |
| Orbital Gyrus | L | 118 | 4.28 | -30 | 50 | -8 |
| Superior Temporal Gyrus | L | 157 | 4.24 | -54 | 0 | -4 |
| Superior Temporal Gyrus | L |  | 3.85 | -56 | -12 | 2 |
| Middle Frontal Gyrus | R | 160 | 4.11 | 38 | 44 | 36 |
| Middle Frontal Gyrus | R |  | 4.04 | 40 | 32 | 34 |
| Middle Frontal Gyrus | R |  | 4.00 | 36 | 48 | 28 |

**Table S7.** Decision-making: Computer-Human.

|  |  |  |  | | MNI coordinates | | |
| --- | --- | --- | --- | --- | --- | --- | --- |
| Region | Laterality | Cluster size K_E_ | | Z | x | y | z |
| Fusiform Gyrus | R | 3359 | | 6.73 | 42 | -52 | -20 |
| Fusiform Gyrus | R |  | | 6.40 | 44 | -44 | -20 |
| Occipital Gyrus | R |  | | 6.18 | 18 | -88 | -4 |
| Occipital Gyrus | L | 1233 | | 6.17 | -14 | -96 | -10 |
| Fusiform Gyrus | L |  | | 6.00 | -44 | -56 | -20 |
| Occipital Gyrus | L |  | | 5.77 | -28 | -96 | -2 |
| Middle Temporal Gyrus | L | 342 | | 5.25 | -60 | -54 | 10 |
| Posterior Superior Temporal Sulcus | L |  | | 4.97 | -60 | -52 | 2 |
| Inferior Temporal Gyrus | L |  | | 3.56 | -64 | -52 | -6 |
| Orbital Gyrus |  | 119 | | 5.06 | 0 | 50 | -6 |
| Middle Frontal Gyrus | R | 750 | | 4.88 | 46 | 14 | 34 |
| Middle Frontal Gyrus | R |  | | 4.68 | 40 | 4 | 32 |
| Superior Temporal Gyrus | R | 445 | | 4.70 | 40 | 24 | -32 |
| Amygdala | R |  | | 4.60 | 16 | -6 | -14 |
| Superior Frontal Gyrus | R | 109 | | 3.85 | 2 | 56 | 34 |
| Superior Frontal Gyrus | R |  | | 3.55 | 10 | 60 | 30 |

**Table S8.** Feedback: Human-Computer.

|  |  |  |  | MNI coordinates | | |
| --- | --- | --- | --- | --- | --- | --- |
| Region | Laterality | Cluster size K_E_ | Z | x | y | z |
| Amygdala | R | 18098 |  | 18 | -8 | -14 |
| Fusiform Gyrus | R |  |  | 42 | -42 | -18 |
| Fusiform Gyrus | R |  |  | 42 | -52 | -20 |
| Orbital Gyrus | R | 642 | 7.59 | 2 | 54 | -10 |
| Orbital Gyrus | R |  | 7.42 | 2 | 38 | -18 |
| Orbital Gyrus | R |  | 6.84 | 4 | 46 | -12 |
| Precuneus | R | 1076 | 6.18 | 2 | -58 | 34 |
| Cingulate Gyrus | R |  | 5.55 | 4 | -52 | 18 |
| Cingulate Gyrus | R |  | 5.15 | 6 | -50 | 28 |
| Middle Temporal Gyrus | L | 338 | 6.02 | -58 | 0 | -14 |
| Superior Temporal Gyrus | L |  | 3.95 | -54 | 16 | -18 |
| Superior Frontal Gyrus | R | 771 | 5.70 | 4 | 56 | 34 |
| Superior Frontal Gyrus | R |  | 5.62 | 6 | 54 | 20 |
| Middle Frontal Gyrus | L | 801 | 5.47 | -42 | 10 | 32 |
| Inferior Frontal Gyrus | L |  | 5.07 | -42 | 16 | 26 |
| Inferior Frontal Gyrus | L |  | 4.61 | -44 | 24 | 20 |

**Table S9.** Feedback: Computer-Human.

|  |  |  |  | MNI coordinates | | |
| --- | --- | --- | --- | --- | --- | --- |
| Region | Laterality | Cluster size K_E_ | Z | x | y | z |
| Cuneus | R | 28488 | 7.65 | 10 | -84 | 22 |
| Cuneus | R |  | 7.32 | 8 | -80 | 32 |
| Superior Frontal Gyrus | L | 640 | 6.23 | -14 | 22 | 60 |
| Superior Frontal Gyrus | L |  | 4.67 | -22 | -6 | 64 |
| Middle Frontal Gyrus | L |  | 4.31 | -20 | 10 | 52 |
| Superior Frontal Gyrus | R | 491 | 5.57 | 20 | 20 | 64 |
| Superior Frontal Gyrus | R |  | 4.14 | 28 | 4 | 62 |
| Superior Frontal Gyrus | R |  | 4.02 | 22 | 2 | 68 |
| Inferior Parietal Lobule | R | 381 | 5.11 | 48 | -44 | 38 |
| Inferior Parietal Lobule | R |  | 4.82 | 56 | -50 | 42 |
| Inferior Parietal Lobule | R |  | 4.60 | 52 | -48 | 50 |
| Precentral Gyrus | R | 780 | 4.92 | 8 | -12 | 72 |
| Superior Frontal Gyrus | R |  | 4.84 | 6 | 6 | 44 |
| Superior Frontal Gyrus | L |  | 4.82 | -14 | -10 | 72 |
| Postcentral Gyrus | R | 163 | 4.47 | 40 | -18 | 40 |

**Table S10.** The brain activity of the interaction between competitor type, others’ choices, and social anxiety.

|  |  |  |  | MNI coordinates | | |
| --- | --- | --- | --- | --- | --- | --- |
| Region | Laterality | Cluster size K_E_ | Z | x | y | z |
| Dorsolateral Prefrontal Cortex | L | 67 | 3.90 | -32 | 32 | 28 |

**Table S11.** Brain functional connectivity of the interaction between others’ choices and social anxiety in the human condition.

|  |  |  |  | MNI coordinates | | |
| --- | --- | --- | --- | --- | --- | --- |
| Region | Laterality | Cluster size K_E_ | Z | x | y | z |
| Inferior Parietal Lobule | L | 127 | 3.96 | -42 | -54 | 40 |
| Inferior Parietal Lobule | L |  | 3.44 | -44 | -60 | 46 |
| Inferior Parietal Lobule | L |  | 3.30 | -48 | -48 | 38 |
